# Beneficial Effects of Macroporous Resin Extract of *Dendrobium candidum* Leaves in Rats with Hyperuricemia Induced by a High-Purine Diet

**DOI:** 10.1155/2020/3086106

**Published:** 2020-02-03

**Authors:** Xiao-Jing Lou, Yu-Zhi Wang, Shan-Shan Lei, Xinglishang He, Ting-Ting Lu, Liang-Hui Zhan, Xue Chen, Ye-Hui Chen, Bo Li, Xiang Zheng, Gui-Yuan Lv, Su-Hong Chen

**Affiliations:** ^1^Collaborative Innovation Center of Yangtze River Delta Region Green Pharmaceuticals, Zhejiang University of Technology, Hangzhou, Zhejiang 310014, China; ^2^College of Pharmaceutical Science, Zhejiang Chinese Medical University, Hangzhou, Zhejiang 310014, China

## Abstract

*Objectives*. The incidence of hyperuricemia (HUA) is increasing year by year, and there are no ideal drugs for the treatment; the existing ones can cause serious liver and kidney damage. We have confirmed that the water extract of *Dendrobium candidum* leaves could reduce the level of uric acid in rats, but the active ingredients remain unknown, and the mechanism is not well understood. This research investigated the therapeutic effect of the macroporous resin extract of the *Dendrobium candidum* leaf (DLE) on hyperuricemia. In this study, hyperuricemia was induced in rats by a 5-week high-purine diet. After that, DLE was administered continuously for 9 weeks. The result showed that biochemical parameters of liver and kidney function, especially serum uric acid (UA) levels, were significantly improved with DLE, which may relate to the reduction of xanthine oxidase (XOD) and adenosine deaminase (ADA) in the liver. Moreover, DLE could significantly prevent kidney and liver from damage, and intestinal injury and reduce inflammation in hyperuricemic rats by inhibiting the expression of both NF-*κ*B and TLR4 proteins. These results showed that the macroporous resin extract of the *Dendrobium candidum* leaves may be effective for the treatment of hyperuricemia in rats by inhibiting uric acid production and decreasing inflammation.

## 1. Introduction

Hyperuricemia (HUA) is a congenital or acquired disease that reduces uric acid excretion, causing an increase in serum uric acid (UA) [1]. The international diagnostic criteria for HUA is that on a normal purine diet after fasting for 2 days, the value of serum UA in men is above 420 *μ*mol/L (7.0 mg/dL) and higher than 357 *μ*mol/L (6.0 mg/dL) in women [2]. A large number of epidemiological and clinical studies have shown that HUA is not only a single metabolic disease but also closely related to many diseases such as hypertension [3], hyperlipidemia, diabetes [4], coronary heart disease [5], arteriosclerosis [6], and obesity. In recent years, with the improvement of living standards, irregular diet, and excessive intake of fat, the prevalence of HUA in China has increased by about 10 times, 7.3% to 58.4% for men and 1.3% to 23.8% for women [7].

The pathogenesis of HUA is complicated. Any factor causing uricogenesis or reduced excretion could lead to HUA [8]. The liver is the main organ for de novo purine synthesis and production of uric acid [9]. Uric acid is the final product of purine catabolism, and a large amount of purine catabolic enzymes is needed during the process. Generally, these enzymes fall into two categories: one is enzymes for uric acid synthesis promotion, including xanthine oxidase (XOD), adenosine deaminase (ADA), guanine deaminase (GDA), and 5-phosphate ribose-1-pyrophosphate synthase (PRS); the other is enzymes inhibiting uric acid synthesis, such as hypoxanthine guanine phosphoribosyl transferase (HGPRT) [10, 11]. Among them, the XOD/ADA system produces a large amount of reactive oxygen species (ROS) while producing uric acid, causing further inflammation of liver and kidney [12]. Uric acid produces a large amount of active oxygen during the synthesis process. Reactive oxygen species not only cause liver and kidney damage but also can be activated by the toll-like receptor 4 (TLR4)/NF-*κ*B signaling pathway, altering the microenvironment of membrane receptors, proteases, and ion channels, leading to increased uric acid production [13].

Drugs such as allopurinol and benzbromarone have long been used to treat hyperuricemia [14]. However, these drugs have long-term adverse effects. Hepatitis and renal function deterioration can occur after long-term use of allopurinol [15].

*Dendrobium officinale* Kimura et Migo belongs to Dendrobium Sw, Orchidaceae and was first described in the Shennong Bencao, a Chinese book on agricultural and medicinal plants published around the 2^nd^ century CE [16]. The Chinese Pharmacopoeia stipulates that the medicinal part of *Dendrobium candidum* is the stem, and the stem is usually processed into a spiral product. But its leaves are often overlooked, and a large number of *Dendrobium candidum* leaves are wasted every year [17]. At present, the research on *Dendrobium candidum* is concentrated on the stem, and little research on the leaf is being carried out. In fact, *Dendrobium candidum* leaves have broad potential for therapeutic use. For example, *Dendrobium candidum* leaves have a good adjuvant effect on hypertension [18], hyperlipidemia [19], inflammation [20], and the like. In our previous studies, we have confirmed that the water extract of *Dendrobium candidum* leaves could reduce the level of uric acid in rats. Although we have studied some of its components [21, 22], its active ingredients are still unknown, and the mechanism is not yet fully understood. To the best of our knowledge, there are no reports in the literature on the treatment of hyperuricemia with extracts of *Dendrobium candidum* leaves.

This study aimed to establish a model of hyperuricemia through a modified purine feed to study the pharmacodynamics of *Dendrobium candidum* leaves and explore the mechanism of antihyperuricemia through the XOD/ADA system and the TLR4/NF-*κ*B inflammatory signaling pathway. The study may lay the foundation for the development of new drugs for hyperuricemia.

## 2. Materials and Methods

### 2.1. Materials and Reagents

*Dendrobium candidum* leaves was purchased from Yunnan Alpine Agriculture Co., Ltd (Yunnan, China). ADS-17 macroporous adsorption resin was purchased from Ganzhou Baoen Adsorption Material Technology Co., Ltd (Hebei, China). Reagent kits such as serum uric acid (UA), creatinine (Cr), urea nitrogen (BUN), aspartate aminotransferase (AST), alanine aminotransferase (ALT), and adenosine deaminase (ADA) were purchased from Ningbo Meikang Biotechnology Co., Ltd. (Zhejiang, China). Hematoxylin-eosin (HE) dye solution and 3,3′-Diaminobenzidine (DAB) chromogenic agent were purchased from Nanjing Jiancheng Technology Co., Ltd. (Jiangsu, China). Modified Masson and periodic acid-schiff (PAS) dye solution were purchased from Shanghai Yuanye Technology Co., Ltd. (Shanghai, China). Rabbit polyclonal antibodies nuclear factor-kappaB (NF-*κ*B), and toll-like receptor 4 (TLR4) were purchased from Huaan Biotechnology Co., Ltd. (Zhejiang, China). HRP-conjugated goat antirabbit IgG was purchased from Beijing Zhongshan Jinqiao Biotechnology Co., Ltd. (Beijing, China).

### 2.2. Macroporous Resin Extract of Dendrobium Candidum Leaves (DLE)

**Figure 1:**
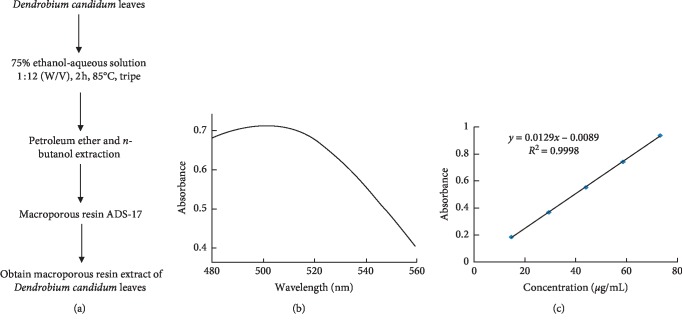
Preparation of macroporous resin extract of *Dendrobium candidum* leaves (DLE). (a) Flowchart for determination of macroporous resin extract. (b) The wavelength of maximum absorption of flavonoids. (c) The rutin standard curve.

*Dendrobium candidum* (DC) leaves are provided by Yunnan Alpine Agriculture Co., Ltd. The preparation of macroporous resin extract of *Dendrobium candidum* leaves (DLE) was done as we described previously (Figure 1) [23]. Briefly, dried *Dendrobium candidum* leaves were extracted three times with 75% ethanol aqueous solution (*v/v*), and then, all the leachates were collected and concentrated under vacuum to acquire the ethanol extract. The petroleum ether and *n*-butanol extraction were successively adopted for the ethanol extract redissolved in aqueous solution. The *n*-butanol fraction was redissolved in aqueous solution for macroporous resin purification. The impurities were first rinsed by deionized water and 10% ethanol aqueous solution (*v/v*); the 50% ethanol aqueous solution (*v/v*) eluent was collected and concentrated under vacuum to prepare the DLE for further animal experiments.

### 2.3. Animals and Experimental Design

Male Sprague Dawley (SD) rats (license number: SCXK 2014-0001) were purchased from Zhejiang Academy of Medical Sciences (Hangzhou China). The housing facility is keeping with the national standards principles of GB14925-2010 (Laboratory Animal-Requirement of Environment and Housing Facilities) for laboratory. The care and experimental operation were conforming to the rules of the “Zhejiang province Administration Rule of Laboratory Aminal.”

SD rats in the model group were given modified high-purine diet (0.15% adenine, 10% yeast extract, and 89.85% standard diet), whereas the normal group was fed with fundamental feed on a daily basis for five weeks. The model group rats were randomly divided into 3 groups (*n* = 10), including model group, low-dose group (4.375 mg/kg body weight, DLE-L), high-dose group (17.5 mg/kg body weight, DLE-H), based on UA (data were not shown), and the high-purine diet was continued during the drug treatment, and potassium oxazinate was given by gavage for 1h before blood sample collection. Then, the corresponding drugs and dosage were given to rats by oral administration. All rats were permitted to drink regular water freely.

### 2.4. Determination of Liver and Kidney Function Biochemical Parameters

Blood samples were taken from the ophthalmic venous plexus during the experiment and were water bathed for 2 hours at 37°C and centrifuged at 3500 rpm for 10 min. The serum was separated to detect the biochemical indexes of UA, Cr, and BUN by an automated biochemical analyzer (Hitachi 7020). At the end of the experiment, the serum was also separated to detect the biochemical indexes of ALT and AST.

### 2.5. Determination of the Fractional Excretion of Uric Acid (FEUA%)

At the 9^th^ week of administration, each group were placed in a metabolic cage fasting for solids and liquids for 24 h, and the urines of each group were collected for FEUA determination; this method was described by Pang et al. [24]. Formula for calculating uric acid excretion score is as follows: FEUA% = uUA ∗ sCr/(sUA ∗ uCr) ∗ 100%. uUA and uCr, uric UA and Cr; sUA and sCr, serum UA and Cr.

### 2.6. Determination of XOD and ADA Activities in Serum and Liver

At the end of the experiment, the rats were fasted overnight and anesthetized with pentobarbital. The liver tissue was weighed, and a volume of saline solution was added and mixed at a ratio (*w/v*) of 1 : 9. Then, the liver samples were homogenized and centrifuged at 12,000 rpm for 10 min at 4°C, and the supernatants were prepared for the assay. The levels of XOD activities in the liver and serum were estimated by rats enzyme-linked immunosorbent assay (ELISA) kits according to operating instructions. The levels of ADA activities in the liver and serum were detected by an automated biochemical analyzer (Hitachi 7020).

### 2.7. Histopathology Observation of Kidney, Liver, and Intestine

Meanwhile, kidney, liver, and intestine were put into 4% formalin for fixation. Then, the organs were cut into applicable slice and went through washing, dehydration, and embedded to make of tissue wax chunks (MEIKO EC360 Tissue Embedder, Germany). All the specimens were cut into 4 *μ*m thickness (LEICARM2245 slicing machine, Germany) and stained by hematoxylin-eosin (H&E). In addition, the specimens of kidney thickness were also used for Masson's trichrome and periodic acid-schiff (PAS) staining. All staining was photographed with the biological microscope (BX43, Olympus, Japan) and analysed with the Image-Pro-Plus 6.0 image analysis software to estimate glycogen deposition, inflammation, and fibrosis lesion.

Semiquantitative analysis was conducted on the proliferation rate of mesangial matrix by PAS staining; kidney tissue of five rats was randomly selected from each group. The complete glomerulus of each slices was observed with a microscope. Statistical analysis was performed on the glomerular mesangial proliferation area/total glomerular area using Image-Pro Plus 6.0 image analysis software.

For the semiquantitative analysis on intestinal villus height (V) and crypt depth (C), kidney tissue of five rats were randomly selected from the ileum H & E-stained slices, and six flattened villi were randomly selected under microscope. Statistical analysis was performed on each group of V, C, and V/C using Image-Pro Plus 6.0 image analysis software.

### 2.8. Immunohistochemistry (IHC) Observation

The immunohistochemistry (IHC) staining was similar as we described previously. The kidney and liver tissues were dewaxed and dehydrated before blocking endogenous peroxidase activity with 0.5% H_2_O_2_ for 10 min. Nonspecific binding was blocked with 10% normal goat serum in PBS for 1 h at room temperature. Subsequently, the sections were incubated with the anti-TLR4 and anti-NF-*κ*B antibody, respectively (1 : 100), in PBS at 4°C overnight. A secondary antibody HRP conjugated goat antirabbit IgG was added. The signals were visualized by DAB staining, and the nuclei were counterstained with hematoxylin. Positive staining presented yellow or brown color under the biological microscope. The data of protein expressions were present by semiquantitative analysis as integrated option density (IOD) in positive area of the microphotograph with the Image-Pro-Plus 6.0 image analysis software.

### 2.9. Statistical Analysis

All the experiment data were expressed as mean ± standard deviation. The data were subjected to one-way analysis of variance (ANOVA). Differences were considered statistically significant if the *p* value was less than 0.05. Diagrams were performed by Graph Prims 7.0.

## 3. Results

### 3.1. Preparation of Macroporous Resin Extract of *Dendrobium candidum* Leaves (DLE)

The content of total flavonoids in dried leaves of *Dendrobium candidum* was 1.16 ± 0.19% as measured by sodium nitrite-aluminum nitrate colorimetry. The purity of the total flavonoids in the *n*-butanol extract obtained by the extraction with the organic reagent was 3.70 ± 0.17%. The purity of total flavonoids in the extract of the total flavonoids purified by ADS-17 macroporous adsorption resin was 18.07 ± 0.03%, and the purity improved by nearly 17 times.

### 3.2. DLE Decreased Serum UA and Prevented Liver and Kidney from Damage in the Hyperuricemic Rats

**Figure 2:**
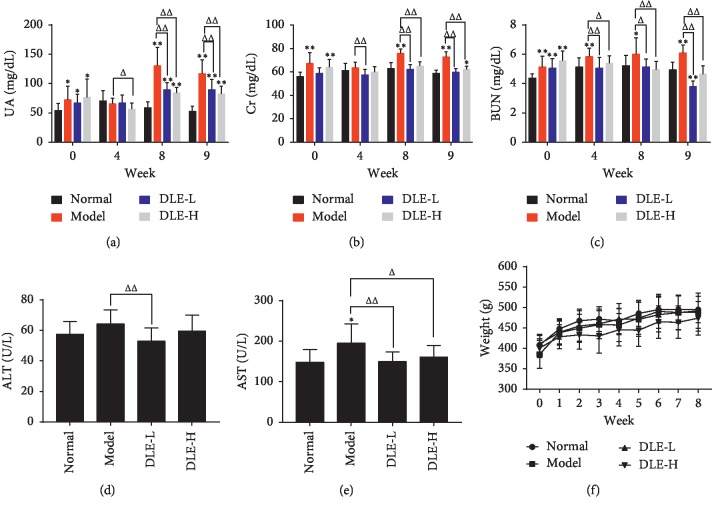
DLE decreased serum UA and prevents liver and kidney damage. (a) Serum uric acid (UA). (b) Serum creatinine (Cr). (c) Serum urea nitrogen (BUN). (d) Serum alanine aminotransferase (ALT). (e) Serum aspartate aminotransferase (AST). (f) Body weight change over time. Normal, the normal control group; Model, the model group; DLE-L, low dose of the macroporous resin extract of *Dendrobium candidum* leaves; DLE-H, high dose of the macroporous resin extract of *Dendrobium candidum* leaves. The data were expressed as mean ± SD (*n* = 9-10). ^*∗∗*^*p* < 0.01 and ^*∗*^*p* < 0.05 versus the normal control group; ^ΔΔ^*p* < 0.01 and ^Δ^*p* < 0.05 versus the model group.

As shown in Figure 2(a)–2(c), at the 8^th^ and 9^th^ week of administration, the DLE of both high- and low-dose groups could stably reduce serum UA and Cr levels compared with the model group (*p* < 0.01). There was a significant difference of serum BUN between the DLE groups and the model group from the 4^th^ week of administration (*p* < 0.05, *p* < 0.01).

AST and ALT are reliable indicators for the assessment of liver function, which is involved in the production of UA [25]. The results were shown in Figures 2(d) and 2(e), the DLE-L could significantly reduce the ALT and AST levels in comparison with the model group (*p* < 0.01), and the DLE-H could significantly reduce the AST level. These results demonstrated that the DLE could prevent the liver from damage in hyperuricemic rats. There was no significant difference in weight gain rate between the normal and model groups. The DLE exerted little effect on weight of the rats (Figure 2(f)).

### 3.3. Effects of DLE on Histopathology of Kidney and Intestine Tissues in Hyperuricemic Rats

**Figure 3:**
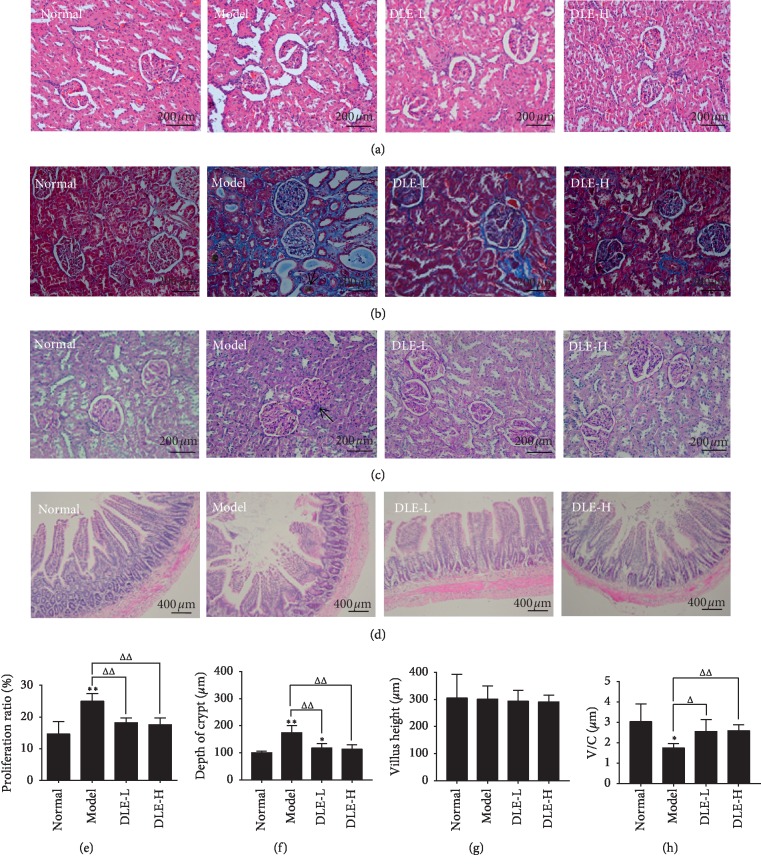
DLE prevents the kidney and intestine histopathology in hyperuricemic rats at the 9^th^ week of administration. (a) Kidney damages directly reflected by hematoxylin-eosin (H & E) (x400). (b) Kidney damages directly reflected by Masson (x400). (c) Kidney damages directly reflected by periodic acid-schiff (PAS) (x 400). (d) Intestinal damages directly reflected by H&E (x200). (e) The data of kidney histopathology was semiquantitatively analysed. Normal, the normal control group; model, the model group; DLE-L, low dose of the macroporous resin extract of *Dendrobium candidum* leaves; DLE-H, high dose of the macroporous resin extract of *Dendrobium candidum* leaves. (f, g, and h) Villus height, depth of crypt, and ratio of villus height to depth of crypt of intestine. Normal, the normal control group; Model, the model group; DLE-L, low dose of the macroporous resin extract of *Dendrobium candidum* leaves; DLE-H, high dose of the macroporous resin extract of *Dendrobium candidum* leaves. The data were expressed as mean ± SD (*n* = 5). ^*∗∗*^*p* < 0.01 and ^*∗*^*p* < 0.05 versus the normal control group; ^ΔΔ^*p* < 0.01 and ^Δ^*p* < 0.05 versus the model group.

Upon H&E staining, compared with the normal group, glomerular cell proliferation and inflammatory cells infiltration were mainly found in the model group, and glomerular extracellular matrix (ECM) accumulation, glomerular mesangial cell proliferation, and basal membrane thickness were observed as well. Both DLE-L and DLE-H could prevent renal injury through alleviating ECM accumulation and inflammatory cell infiltration (Figure 3(a)).

In Masson staining, blue indicates fibrosis. Compared with the normal group, although large areas of blue area were observed in the model group, in addition to the yellow-brown urate crystals (as shown by ↑), the mesangial proliferation is severe, and a large amount of blue collagen fibers are aggregated. DLE can prevent renal interstitial fibrosis and mesangial proliferation of glomeruli (Figure 3(b)).

Upon PAS staining, the glomerular basement membrane of the model group showed remarkable thick ending (as shown by ↑), and a large amount of glycogen deposition and mesangial matrix proliferation were found. We found that the ratio of mesangial matrix proliferation in the model group was significantly increased compared with that in the normal group (*p* < 0.01). The DLE reversed these parameters (*p* < 0.01) (Figures 3(c)–3(e)).

The intestine is involved in uric acid excretion. As shown in Figure 3(d), the villus height (V) of the model group was shorter, the depth of the crypts (C) was larger (*p* < 0.01), and the value of V/C was significantly decreased (*p* < 0.05) compared with the normal group. The DLE could largely decrease C level and increase V/C level compared with the model group (*p* < 0.05, *p* < 0.01) (Figures 3(f)–3(h)).

### 3.4. DLE Decreased Liver XOD and ADA in the Hyperuricemic Rats

**Figure 4:**
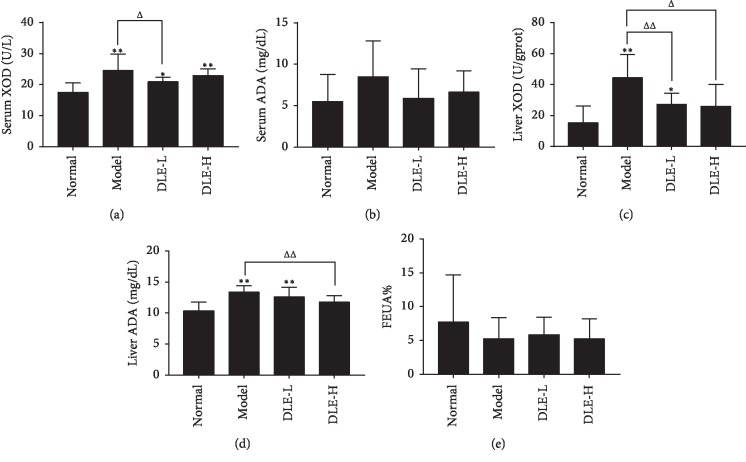
DLE decreased serum and liver XOD and ADA in hyperuricemic rats at the 9th week of administration. (a and b) Serum xanthine oxidase (XOD) and adenosine deaminase (ADA). (c and d) Liver xanthine oxidase (XOD) and adenosine deaminase (ADA). (e) The fractional excretion of uric acid (FEUA%). Normal, the normal control group; Model, the model group; DLE-L, low dose of the macroporous resin extract of *Dendrobium candidum* leaves; DLE-H, high dose of the macroporous resin extract of *Dendrobium candidum* leaves. The data were expressed as mean ± SD (*n* = 9-10). ^*∗∗*^*p* < 0.01 and ^*∗*^*p* < 0.05 versus the normal control group; ^ΔΔ^*p* < 0.01 and ^Δ^*p* < 0.05 versus the model group.

Liver is the main organ for the de novo purine synthesis and the production of uric acid in vivo. XOD and ADA, purine catabolism enzymes, not only in blood but also in liver, are needed during the process. The effect of DLE lowering uric acid may be related to inhibiting uric acid production by acting on ADA and XOD. As shown in Figure 4(a) and 4(b), serum XOD levels in the model group were dramatically higher than that in the normal group (*p* < 0.01), and serum XOD levels in the DLE-L group decreased (*p* < 0.05) compared with the model group. In addition, the DLE-L significantly lowered serum XOD levels (*p* < 0.05). Also, there were no significant differences on ADA in the DLE, the normal, and the model groups. The results of XOD and ADA levels in liver tissue were basically higher than that in the normal group (*p* < 0.01), and the liver XOD and ADA levels in the DLE -H group decreased compared with the model group (*p* < 0.05, 0.01) (Figures 4(c) and 4(d)).

The FEUA% of the model group showed a tendency to decrease in comparison with the normal group. The DLE only slightly increased FEUA% level compared with the model group (Figure 4(e)). It indicated that the DLE has a notable effect on lowering serum UA in hyperuricemic rats induced by modified high-purine diet, but it has no obvious effect on excretion of uric acid. These results demonstrated that the DLE may inhibit the uric acid production in hyperuricemic rats by lowering ADA and XOD in liver.

### 3.5. Effect of DLE on TLR4/NF-*κ*B Expression in Kidney and Liver of the Hyperuricemic Rats

**Figure 5:**
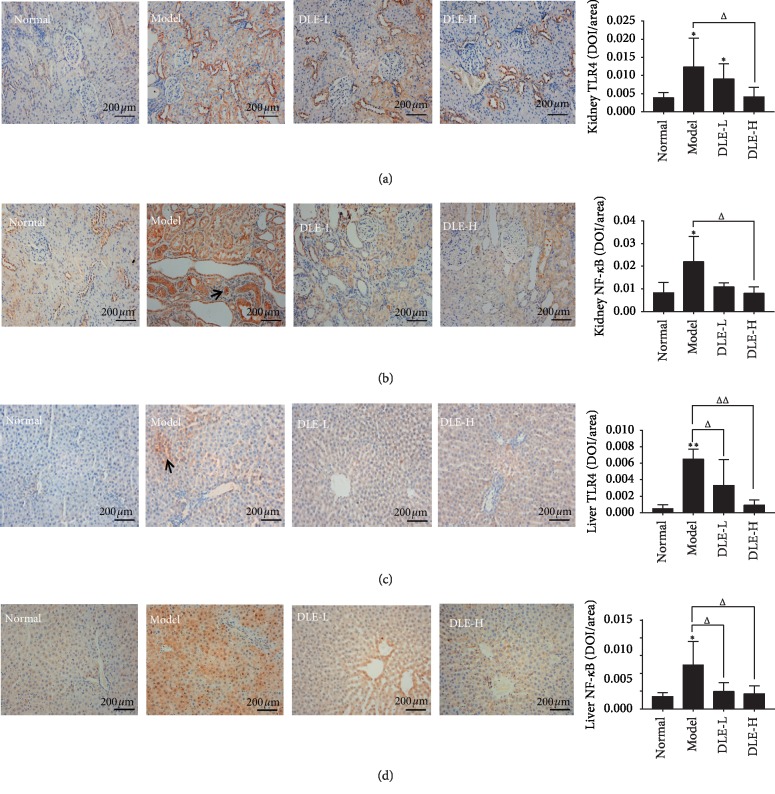
DLE improved TLR4/NF-*κ*B pathway in kidney and liver of the hyperuricemic rats at the 9th week of administration. (a and b) Expression of TLR4 and NF-*κ*B in kidney by immunohistochemistry (ICH) (x400) and semiquantitatively analysed as integrated option density (IOD) in positive area of the microphotograph. (c and d) Expression of TLR4 and NF-*κ*B in kidney by immunohistochemistry (ICH) (x400) and semiquantitatively analysed as integrated option density (IOD) in positive area of the microphotograph. TLR4, toll-like receptor 4; NF-*κ*B, nuclear factor-kappaB; Normal, the normal control group; Model, the model group; DLE-L, low dose of the macroporous resin extract of *Dendrobium candidum* leaves; DLE-H, high dose of the macroporous resin extract of *Dendrobium candidum* leaves. The data were expressed as mean ± SD (*n* = 5). ^*∗∗*^*p* < 0.01 and ^*∗*^*p* < 0.05 versus the normal control group; ^ΔΔ^*p* < 0.01 and ^Δ^*p* < 0.05 versus the model group.

Uric acid can alter the protein expression level of TLR4/NF-*κ*B signaling pathway in kidney. The TLR4 and NF-*κ*B expression was strongly expressed in renal tubular epithelial cells of the model group compared with the normal group (*p* < 0.05) (Figures 5(a) and 5(b)). The DLE-H significantly decreased the TLR4 and NF-*κ*B expression (*p* < 0.05), and this effect was positively related with the dosage, compared with the model group. In Figures 5(c) and 5(d), the TLR4 and NF-*κ*B expression in liver of the model group were strikingly higher than that of the normal group (*p* < 0.01). Compared with the model group, the TLR4 and NF-*κ*B expression in liver of the DLE group was significantly reduced (*p* < 0.05, 0.01).

## 4. Discussion

With people's lifestyle and diet changes in recent years, more seafood and alcohol are being consumed, particularly in economically developed and coastal regions. These foods are rich in purine, protein, nucleotides, and B vitamins [26]. A large amount of yeast and purine could interfere with normal purine metabolism in body, resulting in metabolic disorders such as increasing XOD activity and uric acid production [27].

The average uric acid pool in the human body is 1200 mg; about 750 mg of uric acid is produced per day and approximately 500 to 1000 mg excreted daily (about two-thirds of which is excreted by the kidneys, and the remaining one-third is excreted in the intestine) [28]. The kidney plays an indispensable role in uric acid excretion [29]. The proximal tubule is the site of uric acid reabsorption and secretion, and approximately 90% is reabsorbed into blood [30]. Reabsorption depends on the uric acid transporter on the proximal tubule [31].

Studies have shown that elevated renal uric acid levels can cause histological kidney damage. Elevated UA causes mild tubular interstitial fibrosis, tubular damage, macrophage infiltration, and the appearance of markers of collagen III deposition [32]. UA may also be involved in the progression of liver disease and may eventually contribute to the development of cirrhosis and amplify liver inflammation [33]. Modern pharmacological studies have shown that *Dendrobium candidum* has a certain protective effect against liver and kidney damage [34, 35]. A preliminary study in our laboratory found that *Dendrobium candidum* extract reduces uric acid levels. In fact, the chemical composition of stems and leaves is similar, so it is speculated that the leaves of *Dendrobium candidum* may have corresponding liver and kidney protection and a uric acid–lowering effect. We successfully established a model of hyperuricemia in rats through purine plus yeast feeding and found that the macroporous resin extract of *Dendrobium candidum* (DLE) could effectively reduce UA levels and improve renal function, which was assessed through kidney (CR, BUN) (Figures 2(b) and 2(c)) and liver function indicators (ALT, AST) (Figures 2(d) and 2(e)). Histopathological investigation also shows that DLE can ameliorate liver and kidney damage. In fact, we also found that DLE can prevent pathology of the intestine in rats with hyperuricemia by decreasing the C level and increasing the V/C ratio. These results proved that the DLE could lower uric acid and prevent kidney, liver, and intestinal from damage, which are also associated with an increase in uric acid.

Many enzymes are involved in the process of converting adenine and guanine into uric acid. First, adenosine monophosphate (AMP) is converted to inosine by two different mechanisms. The amino group is first removed by adenine deaminase to form inosine monophosphate (IMP), then dephosphorylated with a nucleotidase to form inosine; the phosphate group is first removed by a nucleotidase to form adenosine, and then adenosine is deaminated by ADA to form inosine. Guanine monophosphate (GMP) is converted to guanosine by nucleotidase. The nucleosides, inosine and guanosine are further converted to the purine bases hypoxanthine and guanine by purine nucleoside phosphorylase (PNP). The hypoxanthine is then oxidized to form xanthine by xanthine oxidase (XOD) and xanthine dehydrogenase (XDH), and the guanine is deaminated by guanine deaminase to form xanthine. Xanthine is again oxidized by xanthine oxidase to form the final product, uric acid [30]. Our previous studies have found that *Dendrobium candidum* Simiao prescription (DSP) has a good uric acid–lowering effect in rats with adenine- and ethambutol-induced hyperuricemia. The study found that DSP can reduce the content of XOD, increase the uric acid excretion index, and increase the level of uric acid transporter [36]. This study found that the DLE could effectively reduce serum XOD levels. In addition, the XOD and ADA levels in the liver of the DLE group were significantly lower than those in the model group. These results indicate that DLE can inhibit uric acid production in hyperuricemic rats by reducing ADA and XOD.

When the enzyme XOD activity is increased, it directly promotes uric acid synthesis and then stimulates oxidative stress and inflammatory injury, which can damage the liver and kidney by triggering the toll-like receptor 4 (TLR4)/NF-*κ*B signaling pathway [37, 38]. NF-*κ*B is located in the TLR4 downstream signaling pathway [39]. The NF-*κ*B signaling cascade is activated in proximal tubular epithelial cells of hypertensive mice with kidney injury [40]. Other studies have also found that TLRs/NF-*κ*B signaling pathway and NLRP3 inflammatory body activation may lead to IL-1*β*–driven inflammatory response, causing hyperuricemia in kidney injury [41]. Through this study, it was found that DLE can significantly reduce the expression levels of TLR4 and NF-kB in liver and kidney and effectively inhibit the inflammatory reaction (Figure 5). DLE may reduce the level of uric acid by reducing the inflammation and pathology of liver and kidney by affecting the TLRs/NF-*κ*B pathway.

In conclusion, it was found in this study that the macroporous resin extract (DLE) of *Dendrobium candidum* leaves is effective for the treatment of hyperuricemia. The DLE decreased serum UA and prevented liver, kidney, and intestinal from damage by suppressing the XOD/ADA system and the TLR4/NF-*κ*B inflammatory signaling pathway. Our findings have potential value in the clinical treatment of hyperuricemia. However, there are some limitations in our study. For example, LPS is a lipopolysaccharide located in the cell wall of Gram-negative bacteria [42], which are produced because of the imbalance of the gut microbiota. As the permeability of the intestinal mucosa increases and the intestinal barrier is impaired, a large number of intracellular LPS are released into the blood [43], which then activate TLRs/NF-*κ*B in the liver and kidney. In this study, DLE was found to prevent the pathology in the intestine, but it is still unknown whether the prevention is related to the inhibition of the gut microbiota-LPS- TLRs/NF-*κ*B axis. Additional study on the active constituents of *Dendrobium candidum* is required, especially including description of the protein and gene level of some of the indicators in the experiment.

## Data Availability

The data used to support the findings of this study are available from the corresponding author upon request.
